# Manganese Enhanced MRI for Use in Studying Neurodegenerative Diseases

**DOI:** 10.3389/fncir.2018.00114

**Published:** 2019-01-07

**Authors:** Galit Saar, Alan P. Koretsky

**Affiliations:** Laboratory of Functional and Molecular Imaging, National Institute of Neurological Disorders and Stroke (NINDS), National Institutes of Health, Bethesda, MD, United States

**Keywords:** manganese, MEMRI, neurodegeneration, neuronal connectivity, tract tracing, manganese-52, molecular imaging

## Abstract

MRI has been extensively used in neurodegenerative disorders, such as Alzheimer’s disease (AD), frontal-temporal dementia (FTD), mild cognitive impairment (MCI), Parkinson’s disease (PD), Huntington’s disease (HD) and amyotrophic lateral sclerosis (ALS). MRI is important for monitoring the neurodegenerative components in other diseases such as epilepsy, stroke and multiple sclerosis (MS). Manganese enhanced MRI (MEMRI) has been used in many preclinical studies to image anatomy and cytoarchitecture, to obtain functional information in areas of the brain and to study neuronal connections. This is due to Mn^2+^ ability to enter excitable cells through voltage gated calcium channels and be actively transported in an anterograde manner along axons and across synapses. The broad range of information obtained from MEMRI has led to the use of Mn^2+^ in many animal models of neurodegeneration which has supplied important insight into brain degeneration in preclinical studies. Here we provide a brief review of MEMRI use in neurodegenerative diseases and in diseases with neurodegenerative components in animal studies and discuss the potential translation of MEMRI to clinical use in the future.

## Introduction

MRI is in widespread use for diagnosis of neurological disorders, for monitoring the progression of disease, response to therapy, and for use in research. MRI is in active development for all diseases that have a neurodegenerative component. This includes the diseases that are primarily neurodegenerative such as Alzheimer’s disease (AD), other forms of dementia such as frontal-temporal dementia (FTD) and mild cognitive impairment (MCI), Parkinson’s disease (PD), Huntington’s disease (HD), and amyotrophic lateral sclerosis (ALS). In addition, MRI is used for the neurodegenerative components of diseases such as epilepsy, stroke and multiple sclerosis (MS) that are not primarily caused by neurodegenerative processes. MRI applications to neurological diseases can be broadly characterized as those where the brain changes have large effects on MRI and can be used for diagnosis, medical decisions and trials with small numbers of participants. These studies rely on well-established MRI contrast mechanisms including enhancement in T_1_ or T_2_ weighted MRI, enhancement with gadolinium-based contrast agents, diffusion/perfusion techniques and fMRI techniques. For example, diffusion MRI is sensitive to early tissue damage due to ischemia which can influence treatment decisions, whereas long-term injury to tissue due to stroke can be measured with T_1_ or T_2_ based MRI contrast (Yoo et al., 2009; Merino and Warach, 2010). Blood brain barrier (BBB) breakage, detected due to leak of gadolinium-based agents, due to inflammation caused by MS is used to quantify the number of active lesions which progress to chronic lesions in T_1_ or T_2_ based MRI after extensive tissue damage (Traboulsee et al., 2016; Reich et al., 2018). MRI contrast has been useful for localizing sites of tissue damage, and to guide surgery for epilepsy, while fMRI has been used to map functional areas such as language to guide these surgeries (Duncan et al., 2016).

MRI has been less successful for diagnosis or treatment decisions in individuals for most of the primarily neurodegenerative diseases. In these cases, MRI has been extremely useful for research or as a way to decrease number of patients required in large trials where MRI can be an additional biomarker. Quantitative anatomical studies have been most useful in these cases. Hippocampal atrophy and ventricular enlargement have been associated with AD and MCI (Jack et al., 2004; Thompson et al., 2004; Ridha et al., 2008; Tang et al., 2014). Cortical thinning has been demonstrated by MRI in a number of neurodegenerative disorders such as HD (Nopoulos et al., 2010; Nanetti et al., 2018). The growing sensitivity of MRI due to increasing field strength and better detectors is allowing studies to be done at resolutions below 0.125 mm^3^ (0.5 mm linear resolution). Smaller volumes of hippocampal subfields such as the subiculum, CA1, CA3 and dentate gyrus (DG), have been measured in Alzheimer’s patients compared to MCI and healthy patients (Kerchner et al., 2010; Mueller et al., 2010; Wisse et al., 2014). Significant reduction in the olfactory bulb (OB) have also been reported in AD and PD patients (Thomann et al., 2009; Wang et al., 2011). In PD, changes in substantia nigra (SN) due to neurodegeneration have been reported (Kwon et al., 2012; Lehéricy et al., 2014) and changes in the striatum volume have been studied in HD (Ross et al., 2014). In patients with ALS, high resolution MRI has detected anatomical changes in primary motor cortex and cortical spinal tract (Cosottini et al., 2016). While these high-resolution studies offer tremendous potential for moving to analyzing small numbers of patients the effect sizes have not been large enough to enable MRI to contribute to diagnosis and response to therapy in individuals. Therefore, there is a need to develop other strategies to enable MRI to detect processes associated with neurodegeneration. Manganese enhanced MRI (MEMRI) has now been used in a large number of preclinical studies in animal models of neurodegeneration. Here we present a brief review of this work and discuss the prospects for translating this type of contrast to humans.

MEMRI has been used to image anatomy and cytoarchitecture, obtain functional information from different areas of the brain, and to trace neural connections and axonal transport rates. All of this relies on the fact that Mn^2+^ is essential for brain health and on the rich and complex biology that the brain uses to move Mn^2+^. Brain architecture at the level of cytoarchitecture has been imaged with MRI including layer specific accumulation in the OB, cortex, hippocampus, retina, and cerebellum after systemic administration (Watanabe et al., 2002; Aoki et al., 2004; Lee et al., 2005; Berkowitz et al., 2006; Silva et al., 2008). Clear cytoarchitectural boundaries for different brain areas have been identified using MEMRI. Under the right conditions increased neural activity in a specific area can lead to increased accumulation of Mn^2+^ and contrast on MRI. Sensory, motor, auditory, hypothalamic, and hippocampal activity have been reported from MEMRI (Lin and Koretsky, 1997; Aoki et al., 2002; Morita et al., 2002; Hsu et al., 2007; Yu et al., 2008; Eschenko et al., 2010; Hankir et al., 2012). A third type of information can be obtained from direct injection of Mn^2+^ into specific areas of the brain where Mn^2+^ will move in an anterograde direction along the direction of information flow to enable neuronal tracing studies (Pautler et al., 1998). Mn^2+^ will move transsynaptically allowing circuits to be mapped. With direct application strategies, MEMRI has been used to trace sensory pathways such as olfactory, visual, somatosensory, and auditory pathways. This can be done at the level of specific cytoarchitectural elements such as individual olfactory glomeruli, or specific cortical laminae (Pautler et al., 1998; Watanabe et al., 2001; Van der Linden et al., 2002; Allegrini and Wiessner, 2003; Leergaard et al., 2003; Cross et al., 2004; Murayama et al., 2006; Canals et al., 2008; Chuang and Koretsky, 2009; Tucciarone et al., 2009). Other pathways such as descending motor pathways and basal ganglia (BG)-striatal pathways have been imaged as well (Saleem et al., 2002; Pautler et al., 2003; Murayama et al., 2006). For detailed review on MEMRI procedures please see Silva et al. (2004). This broad range of information from MEMRI has led to its application in animal models of neurodegeneration, supplying important information about brain degeneration in preclinical studies.

## MEMRI in Animal Models of Neurodegeneration

### Studies Using Systemic Administration of Manganese

Animal models of neurodegenerative diseases have been used in many MEMRI studies, that were mainly focused on either anatomy and cytoarchitecture changes that relates to disease symptoms and pathology. Following systemic administration of manganese, contrast in the brain is achieved 24 h later (Aoki et al., 2004; Lee et al., 2005). This allows the detection of anatomical and laminar changes in the brain to assess degeneration.

In AD, large scale neuronal loss due to amyloid-β (Aβ) plaque formation and the presence of neurofibrillary tangles (NFTs) occurs at late stages of the disease (Hardy and Selkoe, 2002). Animal models of AD are based on overexpression of either amyloid precursor protein (APP) or tau protein that leads to the formation of Aβ plaques or NFTs, respectively. The rTg4510 mouse model of tauopathy, that express high levels of human tau and accumulates NFTs, is a model of both AD and FTD. Following systemic administration of Mn^2+^, impaired accumulation of Mn^2+^ was measured in the total hippocampus and its subregions, and the amygdala. These are structures that relate to memory formation deficits that were detected at early stages of tau formation (3 months of age; Fontaine et al., 2017). The Mn^2+^ accumulation in these regions was further reduced with increased tau pathology at older ages (5–10 months of age; Perez et al., 2013; Fontaine et al., 2017). The 5XFAD mouse model that overexpress APP and presenilin 1, showed increased signal intensity in the hippocampus at early stages of AD (2–5 months of age) compared to controls, while behavioral assays showed increased learning and memory impairment with mice age (Tang et al., 2016). MEMRI results are also consistent with glucose studies in AD mouse models where impaired uptake of glucose (either decreased or increased uptake) was reported at different ages in mice (Luo et al., 2012; Poisnel et al., 2012; Macdonald et al., 2014). EEG studies have detected abnormal spike activity in the hippocampus of AD mice prior to memory impairments, which may explain the underlying basis of MEMRI results (Palop et al., 2007; Kam et al., 2016). Indeed, the increased accumulation of Mn^2+^ in the 5XFAD mouse model at the early stages of AD, may be related to these EEG changes. However, the manganese accumulation mechanisms are not yet fully understood, and it could be due to Mn^2+^ uptake with inflammation as there is a report of increased Mn^2+^ uptake with elevated inflammation in stroke (Kawai et al., 2010).

**Figure 1:**
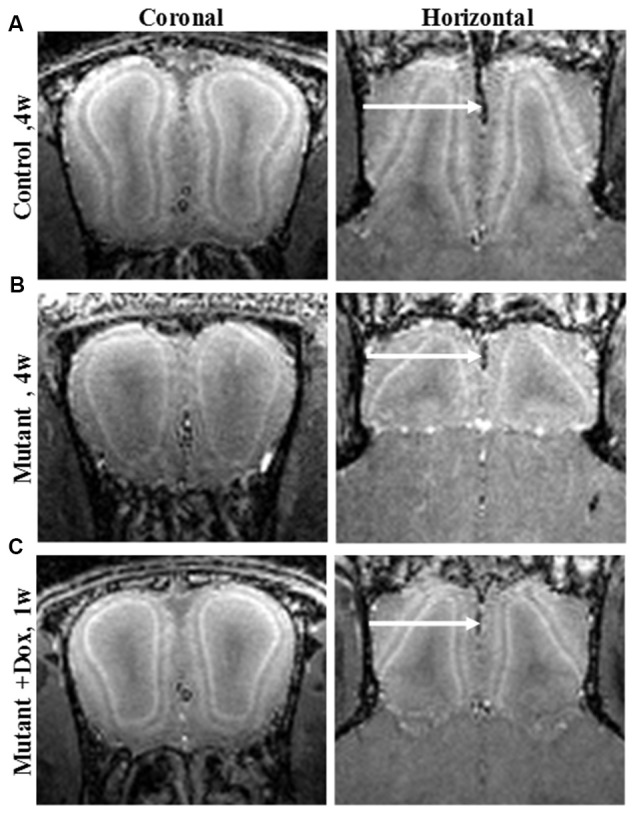
Coronal and horizontal T_1_ weighted images manganese enhanced MRI (MEMRI), at 50 μm isotropic resolution, taken 24 h after iv infusion of 100 mM MnCl_2_ solution of 4-week-old **(A)** control, **(B)** mutant and **(C)** mutant mouse after 1 week of doxycycline treatment mice. Reprinted from Saar et al. (2015) with permission from Elsevier.

MEMRI also enables direct visualization of laminar specific neurodegeneration and its recovery with treatment. Olfactory dysfunction is an early symptom of AD (and other neurodegenerative diseases; Bacon et al., 1998). MEMRI study of an olfactory-based AD mouse model with overexpression of APP specifically in olfactory neurons was shown to detect laminar changes in the OB and this was used to follow neurodegeneration and recovery following systemic administration of manganese (Saar et al., 2015). In the OB, overexpression of humanized-APP resulted in the disruption of the bulb’s laminar structure. There was decreased manganese enhancement in the glomerular layer and a decreased OB volume compared to control (Figures 1A,B). Turning off APP overexpression with doxycycline showed a significant increase in manganese enhancement of the glomerular layer after only 1 week (Figure 1C), with further recovery after 3 weeks of the bulb back to control.

PD is characterized by the loss of dopaminergic neurons in the pars compacta of the SNc that leads to activity changes in the BG nuclei. In animal models of PD, 1-methyl-4-phenyl-1,2,3,6-tetrahydropyridine (MPTP) induces death of dopaminergic neurons in the SNc. Systemic administration of manganese in a PD rat model, 2 weeks after MPTP lesioning in the SNc was induced, resulted in decreased Mn^2+^ accumulation in the SNc and striatum, and hippocampal subregions compared to control rats due to degeneration of dopaminergic neurons. Increased accumulation was measured in the subthalamic nucleus (STN). Treatment of MPTP rats with ceftriaxone, was shown to prevent these changes (Weng et al., 2016). In studies with a PD mouse model of MPTP intoxication, Mn^2+^ enhancement was decreased in the SNc compared to control while the striatum showed increased Mn^2+^ enhancement after 2 days (Olson et al., 2016) and 1 or 2 weeks (Kikuta et al., 2015). In the striatum, the increased Mn^2+^ uptake, in the first few days after MPTP intoxication, was suggested to reflect high astroglial reactivity due to early striatum termini degeneration, which leads to increased signal enhancement (Olson et al., 2016). Thus, Mn^2+^ accumulation could be very dependent on timing with respect to cell death. Treatment with LBT-3627 (a VIPR2 agonist), prior to MPTP intoxication did not result in increased manganese enhancement in the SNc (Olson et al., 2016). These studies show that MEMRI is able to detect neurodegeneration but also enabled testing different reagents ability to block and/or reverse neuronal loss.

Systemic administration of manganese was also used to study neurodegenerative changes in animal models of retinal degeneration. Dark adapted royal college of surgeons (RCS) rats, a model of photoreceptor degeneration, underwent MEMRI before degeneration onset [developmental stage Postnatal day (P) 17] and during the course of degeneration, (P36 and P57, after the loss of rods and cones, respectively). The retinal thickness was significantly reduced only in the pathological stage, P57 rats, compared to age matched control rats. Decreased Mn^2+^ uptake was evident in the developmental stage (P17), prior to the onset of photoreceptor damage, throughout the retina, but increased at later ages. This was attributed to ionic dysregulation during pathological retinal thinning (Berkowitz et al., 2008).

### Neuronal Tracing Studies With MEMRI

Direct injection of manganese into specific brain areas can be used to trace neuronal connections in the brain and allows for the detection of impaired intracellular transport. Thus, MEMRI can be used to study axonal transport and measure axonal transport rates in the rodent brain. In the Tg2576 AD mouse model, Aβ depositions were detected in the OB prior to other brain areas, which resulted in decreased olfactory function (Wesson et al., 2010). Intranasal administration of manganese showed that the axonal transport rates from the olfactory epithelium to the OB layers were decreased prior to Aβ plaque formation and continued to decrease with the progression of Aβ plaque formation in this mouse model (Smith et al., 2007; Wang et al., 2012). Similar results were obtained with an APP knockout (APP^−/−^) mouse model (Smith et al., 2010). A MEMRI study in a triple transgenic AD mouse model, reported decreased axonal transport in the olfactory system, that preceded Aβ plaque deposition and also the formation of NFTs (Kim et al., 2011). In the APP knockout (APP^−/−^) mouse model, Mn^2+^ injection into CA3 region of the hippocampus, showed reduced Mn^2+^ transport from the hippocampus to the amygdala and basal forebrain and to the contralateral hippocampus (Gallagher et al., 2012). Intraocular injection of Mn^2+^ also revealed reduced Mn^2+^ transport in the visual system of those mice (Gallagher et al., 2012). Similar study of Mn^2+^ injection into the CA3 region of the hippocampus in APPSwInd transgenic mice, that express mutant APP with both Swedish and Indiana mutations, showed decreased Mn^2+^ transport along the hippocampus to basal forebrain pathway with ageing and altered Mn^2+^ accumulation in APPSwInd mice that displayed Aβ plaques. This suggest that the natural alteration in neuronal connections with ageing is further disrupted with APP overexpression and the formation of Aβ plaques (Bearer et al., 2018). In addition, MEMRI was used to assess therapeutic reagents and test their ability to improve axonal transport. Treatment with R-Flurbiprofen, that selectively reduce Aβ42, and chronic treatment with MRK-560, a gamma-secretase inhibitor, were shown to significantly improve axonal transport rates in Tg2576 mice (Smith et al., 2011; Wang et al., 2012).

In the rTg4510 mouse model of tauopathy, nasal administration of Mn^2+^ revealed axonal transport deficits in the OB in an age dependent manner starting at 3 months of age, prior to tau pathology (Majid et al., 2014). This is similar to other AD mouse models where axonal transport deficits preceded Aβ plaque formation in the brain. Moreover, a different mouse model of tauopathy, JNPL3, also measured impaired axonal transport of Mn^2+^ with increasing tau pathology (Bertrand et al., 2013).

Axonal transport of manganese was studied in a widely used ALS mouse model, the Tg SOD1-G93A, which is a model of severe neurodegeneration with selective loss of motor neurons and progressive motor weakness. Intranasal administration of manganese showed significantly slower transport rates in OB of ALS mice compared to control mice. Acute treatment with NAP (davunetide), a microtubule interacting compound that protects against tau pathology, resulted in similar axonal transport rates as for control mice in this mouse model of ALS (Jouroukhin et al., 2013).

Intraocular injection of manganese was used to follow retinal degeneration changes of P90 RCS rats, in which photoreceptors are already degenerated. Changes in the retina laminar structure were detected using MEMRI with the loss of three out of the seven layers detected in normal retina as well as significant retinal thinning (Nair et al., 2011).

MEMRI tracing after direct injection was also done in the unilateral 6-hydroxydopamine (6-OHDA) rat model of PD, where lesions are induced in the SNc. Mn^2+^ injection into the globus pallidus nucleus and SN of unilateral 6-OHDA showed increased transport across hemispheres in several structures such as the habenular complex (Hab), and the thalamic anteroventral compared to control rats (Pelled et al., 2007). This suggests a large degree of plasticity in this model of neurodegeneration. A later study used MEMRI to study the etiology of depression in PD with 6-OHDA rat model. Manganese injection to the raphe nuclei showed reduced raphe connectivity, following the loss of dopamine cells and enhanced connectivity in Hab, that was associated with depression like behavior. Apomorphine treatment, a dopamine replacement therapy, resulted in partial recovery in raphe connectivity (Sourani et al., 2012). In contrast to the previous studies, manganese injection into the STN of 6-OHDA rats in a different study, showed decreased axonal transport from ipsilateral STN to structures of BG, such as ventral pallidum compared to control rats (Soria et al., 2011).

Genetic mutations are associated with axonal transport impairment that is commonly found in neurodegenerative diseases, such as APP and tau in AD, SOD1 in ALS, huntingtin in HD and Parkin in PD (Millecamps and Julien, 2013). Indeed, decreased axonal transport rates in affected areas of the brain is a robust finding across a number of animal models of neurodegenerative diseases presented here. Axonal transport measurements using MEMRI gives unique information and would be an ambitious target for potential translation to humans.

## MEMRI in Diseases With Neurodegenerative Components

Although not defined as neurodegenerative diseases, MS, stroke, epilepsy, and glaucoma cause neurodegeneration in later stages. MS is an inflammatory disease of the central nervous system (CNS) and is characterized with lesions that can appear throughout the brain and in later stages with demyelinated axons and progressive neurodegeneration. In stroke, following the acute stage where an ischemic core is formed, the later stages are characterized with ongoing vascular impairment and neurodegeneration. Epilepsy is characterized by spontaneous recurrent seizures with the development of hippocampal sclerosis, neuronal cell loss, inflammation and neurodegeneration.

The most common type of stroke is the ischemic stroke. Animal models of ischemic stroke includes the middle cerebral artery occlusion (MCAO) and photothrombotic cortical injury (PCI). Systemic administration of manganese in rats and mice that undergo unilateral MCAO and PCI showed increased manganese accumulation in the perilesional tissue and reduced Mn^2+^ transport in the first few days after stroke was induced (Aoki et al., 2003; Hao et al., 2016; Chan et al., 2017). The increased Mn^2+^ uptake was attributed to inflammatory processes in the perilesional tissue (Kawai et al., 2010; Hao et al., 2016). This opens the possibility of using Mn^2+^ to measure inflammatory responses. It was also shown that the affected brain area with enhanced Mn^2+^ was smaller than the area that was detected with reduced apparent diffusion coefficient in diffusion MRI. This was interpreted to mean that MEMRI detects the ischemic core consistent with accumulation of Mn^2+^ being due to inflammation (Kawai et al., 2010).

Local injection of manganese into the brain was used to study connectivity in animal models of stroke. Manganese injection into sensorimotor cortex, 2 weeks after unilateral stroke induced by MCAO in rats, exhibited reduced and delayed manganese enhancement in the ipsilateral thalamus and SN which indicates loss of connectivity of areas in the sensorimotor cortex (van der Zijden et al., 2007, 2008; Hao et al., 2016). At later time points, 4 and 10 weeks after stroke, manganese enhancement was restored in ipsilateral sensorimotor cortex and increased enhancement was detected in the contralateral hemisphere that was correlated to functional recovery observed in behavioral tests (van der Zijden et al., 2008). The accumulation in the contralateral hemisphere was attributed to increased plasticity along the corpus callosum (CC). However, manganese injection into the contralateral primary motor cortex showed declined manganese enhancement into the ipsilateral sensorimotor cortex 10 weeks after stroke (van Meer et al., 2010). This result was explained by larger induced lesions and the different location of manganese injection in this study as compared to the earlier study.

MEMRI has also been used in animal models of MS. A common animal model for MS is experimental autoimmune encephalomyelitis (EAE), which uses active immunization with CNS homogenates, myelin, or myelin-derived antigens, that induce autoimmune mediated responses. As optic neuritis is one of the first symptoms of MS, MEMRI was used to assess axonal transport in the optic nerve (ON) in EAE animal models. In an EAE rat model, systemic administration of Mn^2+^ showed increased signal enhancement in the ON compared to control rats 24 h after administration, that was correlated with the severity of axonal loss. The increase in Mn^2+^ might have been due to overload of intracellular Ca^2+^ in response to axonal damage in the ON (Boretius et al., 2008). In an EAE mouse model, following intraocular injection, Mn^2+^ accumulation as well as axonal transport rates were significantly decreased both in moderate and severe optic neuritis when compared to control mice. The degree of alteration in axonal transport was correlated to the extent of visual impairment and changes in axonal pathology (Lin et al., 2014). In another study, the EAE mouse model was used to follow changes in CC connectivity with MEMRI, as atrophy of CC is also observed in MS patients. Manganese was directly injected into the visual cortex and the CC was traced over time. An increase in manganese enhancement was detected in the CC compared to control mice in the first 14 h after Mn^2+^ injection in this study (Chen et al., 2008). It is not clear why an increase was detected in the CC, but it was suggested that it involved ion dyshomeostasis, due to increased intracellular Ca^2+^ accumulation.

Animal models of temporal lobe epilepsy includes the kainic acid (KA) and pilocarpine induced status epilepticus (SE) models that represent the acute phase of epilepsy. In the latent phase, animals are seizure free until the onset of spontaneous recurrent seizure in the chronic phase. These phases are associated with different neurobiological changes such as, hippocampal sclerosis, mossy fiber sprouting, inflammation and neurodegeneration. MEMRI studies after systemic administration of Mn^2+^, showed decreased signal intensity in the hippocampus that was attributed to decreased neuronal activity in a KA rat model (Alvestad et al., 2007; Immonen et al., 2008). Decreased manganese signal in the DG and CA3 was also detected in the acute phase of 30 min induced SE in the pilocarpine model, with the low signal related to edema and not to cell death (Malheiros et al., 2014). Increased manganese signal intensity in the DG and CA3 was detected later in the latent phase (Alvestad et al., 2007). In the chronic phase the epileptic rats showed increased signal intensity in the CA3 and DG compared to control rats. However, this increased MEMRI signal was correlated with mossy fiber sprouting and not with neurodegeneration (Immonen et al., 2008; Malheiros et al., 2012) and was not dependent on seizure frequency (Immonen et al., 2008). In a different rat model, where SE was induced by lithium-pilocarpine injection, MEMRI was used to assess mesenchymal stem cells (MSCs) treatment to reduce epileptogenesis in the hippocampus. In this study, higher manganese signal was detected in the DG and CA3 of vehicle treated epileptic rats compared to controls following systemic Mn^2+^ administration. The manganese enhancement was reduced in MSC infused rats and was associated with suppression of mossy fiber sprouting seen by histology in the MSC group (Fukumura et al., 2018).

In a MEMRI study of neuronal activation, increased manganese signal intensity was observed in the CA3 region of the hippocampus in the acute phase of KA-treated rats compared to control. Injection of diltiazem, an L-type calcium channel blocker, to KA-treated rats, resulted in attenuation of the manganese signal in the CA3 demonstrating an activity dependent uptake. This was correlated with decreased focal edema and decreased neuronal swelling observed by histology following diltiazem injection (Hsu et al., 2007). Direct injection of manganese into the entorhinal cortex of KA injected rats resulted in increased number of manganese enhanced pixels in the DG and CA3 compared to control rats. This increase was correlated to histological mossy fiber sprouting (Nairismägi et al., 2006). Direct Mn^2+^ injection into the lateral ventricle also resulted in increased manganese enhancement in the hippocampus, CA1 and DG, but was inversely correlated with seizure frequency (Dedeurwaerdere et al., 2013).

Glaucoma is characterized by progressive degeneration of retinal ganglion cells, that later effect structures along the visual pathway in the brain. Although not primarily a neurodegenerative disease, its main clinical impact is due to neurodegeneration. The DBA/2J mouse model is a late onset, hereditary glaucoma model associated with age dependent increase in intraocular pressure. MEMRI studies with DBA/2J mice showed changes in ocular anatomy due to glaucoma (Calkins et al., 2008) and decreased manganese enhancement in superior colliculus, ON and lateral geniculate nucleus compared to control mice that was further decreased with age (Fiedorowicz et al., 2018; Yang et al., 2018). Similar results were obtained in an induced ocular hypertension rat model of chronic glaucoma, where decreased Mn^2+^ enhancement in the ON of the glaucomatous eye was observed after intravitreal injection (Chan et al., 2008).

## Translating MEMRI to Humans

The large number of studies that have used Mn^2+^ as a contrast agent in neurodegenerative diseases to assess both anatomical and neuronal connectivity changes in preclinical studies, makes it interesting to determine if it may be possible to apply MEMRI to humans. Unfortunately, the potential use of Mn^2+^ as a contrast agent in human studies of neurodegeneration is limited by toxicity. Most of the animal studies are done at doses that might be hard to justify in humans (Chandra and Shukla, 1976; Wolf and Baum, 1983; Crossgrove and Zheng, 2004). In the past, mangafodipir (MnDPDP, Teslascan), an FDA-approved chelated Mn^2+^ contrast agent was used in clinical studies to image healthy volunteers and patients with liver metastases, pancreatic cancer, and myocardial infarction (Wang et al., 1997; Federle et al., 2000; Schima et al., 2002; Skjold et al., 2007). The specific tissue contrast is due to release of Mn^2+^ from the chelate due to transmetalation with zinc in the blood. However, the FDA-approved label indication for liver metastases detection did not warrant further production and it is not widely available. Furthermore, FDA-approved doses are lower than used in animal studies of neurodegeneration. Nevertheless, animal studies have shown the potential usefulness of MnDPDP to study retinal degeneration (Olsen et al., 2008; Tofts et al., 2010). Unlike gadolinium-based agents, which are extracellular agents with rapid clearance that requires significant extracellular space to detect, the manganese signal enhancement is due to intracellular accumulation of Mn^2+^ that lasts in the tissue long after it is cleared from the blood. This property provides the unique contrast in the brain where cytoarchitecture, activity and neuronal tracing can be detected. Early studies with MnDPDP administration to healthy volunteers, showed increased Mn^2+^ enhancement in the choroid plexus and pituitary gland, structures that lack a BBB (Wang et al., 1997; Sudarshana et al., 2018). It will be interesting to see if MnDPDP enhances the brain in human studies in cases where there is breakdown of the BBB, due to MS and other disorders.

Parenteral nutrition contains manganese, making this a possible way to administer Mn^2+^. Signal enhancement in BG structures, mainly the globus pallidus, in T_1_ weighted images was reported. However, in these cases the patients received long-term parenteral nutrition (>1 month), showed very high whole blood manganese concentrations and in some cases developed parkinsonian-like symptoms. No signal change was detected in the brain of control patients and low manganese exposure patients (Quaghebeur et al., 1996; Aschner et al., 2015; Livingstone, 2018). With these limitations, parenteral nutrition may be useful for some clinical applications of MEMRI. In the past, MnCl_2_ solution was given intravenously to healthy volunteers to study its safety and efficacy for cardiovascular imaging. Decreased myocardium T_1_ values were measured while no adverse events and good tolerance for MnCl_2_ were reported (Fernandes et al., 2011). This may allow the use of MnCl_2_ solution, at appropriate doses, for brain imaging in clinical studies.

Another approach would be to use radioactive Mn^2+^ to enable positron emission tomography (PET) studies rather than rely on MRI. Recent advances in preparation of high energy radiotracers allow for the production of two manganese radiotracers for PET imaging; manganese-51 (^51^Mn) and manganese-52 (^52^Mn) with half-lifes of 46 min and 5.6 days, respectively. As PET has higher sensitivity than MRI, a lower concentration of Mn^2+^ can be used for PET, that may enable its use in clinical studies to obtain information that has been shown to be useful in preclinical MEMRI studies. Indeed, manganese-54, a gamma-emitter (^54^Mn, *t*_1/2_ = 312.5 days) has been used to study manganese accumulation of tissues in the body of mice and monkeys (Dastur et al., 1971; Lydén et al., 1983) and has been used to visualize specific neuronal connections in rats (Sloot and Gramsbergen, 1994; Tjälve et al., 1996; Takeda et al., 1998). Due to its high gamma energy and long half-life (~1 year), ^54^Mn is not likely to be clinically useful.

The half-life of 5.6 days, of the positron emitter ^52^Mn allows its use in longitudinal biodistribution studies in the body and brain (Topping et al., 2013; Graves et al., 2015; Brunnquell et al., 2016; Hernandez et al., 2017; Napieczynska et al., 2017; Saar et al., 2018). MEMRI studies have shown that following systemic administration, the final distribution of manganese in the brain that allows anatomical structures was achieved 24 h after infusion. This makes ^52^Mn a suitable radiotracer for imaging of brain anatomy and function. ^52^Mn^2+^ infusion in rats showed manganese accumulation of the radiotracer in the head and brain with a similar contrast to that seen in MEMRI (Brunnquell et al., 2016; Saar et al., 2018).

**Figure 2:**
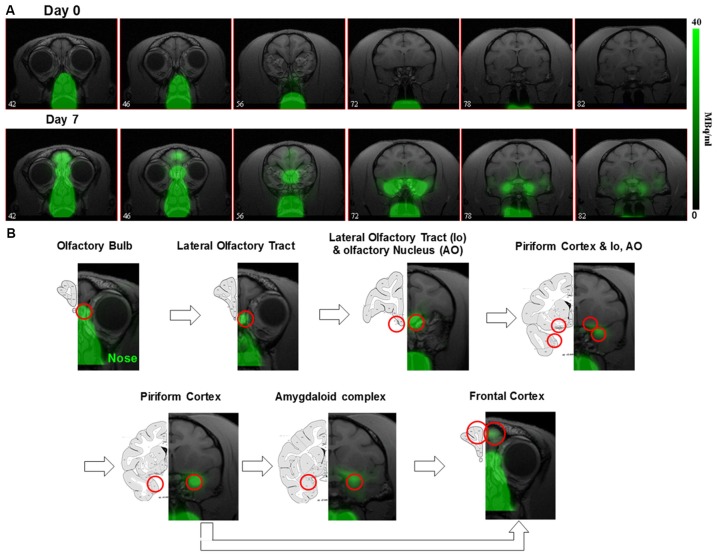
^52^Mn nasal administration in monkeys **(A)** positron emission tomography (PET) images co-registered with MRI images from front to back immediately after (day 0) and 7 days after nasal administration of ^52^Mn^2+^. ^52^Mn^2+^ solution of 7-22 MBq (0.2–0.6 mCi) was administered to both nostrils on day 0 (total volume 0.5 ml). Immediately after ^52^Mn^2+^ administration the radioactivity is localized only to the nose area. By day 7 the ^52^Mn^2+^ traced into the brain from the nose to the amygdala. **(B)** Olfactory pathway in monkeys. PET images co-registered with MRI images following ^52^Mn^2+^ administration into the nostrils and the corresponding monkey atlas images (Martin and Bowden, 2000). ^52^Mn^2+^ administration to the nostrils traces the olfactory pathway from the nose to the olfactory bulb (OB), then to the olfactory tract, olfactory nucleus, piriform cortex, amygdala and the frontal cortex. Reprinted from Saar et al. (2018) with permission from Springer.

The ~6 days half-life of ^52^Mn should also allow neuronal tracing studies. Intranasal administration of ^52^Mn^2+^ traces the olfactory pathway over the course of several days in monkeys (Figure 2; Saar et al., 2018) similar to MEMRI (Pautler et al., 1998; Chuang and Koretsky, 2009). Immediately after administration ^52^Mn^2+^ radioactivity was localized in the nasal turbinates with no manganese accumulation in the brain. By 4 days later ^52^Mn^2+^ could be traced to the amygdala and prefrontal areas of the cortex (Figure 2). Nepieczynska et al (Napieczynska et al., 2017) showed imaging of the dopaminergic and striatonigral pathways in rats 24 h after direct injection of ^52^Mn^2+^ into the ventral tegmental area and the dorsal striatum, respectively. The ability of ^52^Mn^2+^ to follow neuronal pathways after direct injection of the ^52^Mn^2+^ PET tracer to a specific area in the brain similar to MEMRI, can be used to study all the neuronal pathways already studied with MEMRI such as the visual and BG pathways (Pautler et al., 1998; Watanabe et al., 2001; Murayama et al., 2006). Quantification of the axonal transport changes as has been done in MEMRI for animal models of neurodegenerative diseases should also be possible. Finally, the rise in usefulness of focused ultrasound to break the BBB in human should enable delivery of Mn^2+^ to any area of the human brain, as has already been shown in rodents (Howles et al., 2010). It is interesting that the manganese radiotracer ^52^Mn^2+^ for PET imaging acts so similar to that of Mn^2+^ in MEMRI, even though the PET dose is ~2,000-fold lower (Saar et al., 2018). The lower PET resolution may present a problem for using Mn-PET instead of MEMRI as it limits the ability to detect cytoarchitecture and small structures in the brain. However, the growing interest in PET/MRI systems may enable the use of Mn-PET with high resolution MRI image registration. These results demonstrates that ^52^Mn^2+^ may be useful in human studies for imaging neurodegeneration by tracking changes in neuronal connections and anatomical changes at clinically safe doses.

## Conclusion

In conclusion, there is a large growth of preclinical literature that has used MEMRI to study different aspects of neurodegeneration. Due to the wide variety of mechanisms that the brain uses for accumulation of Mn^2+^, care must be taken in assigning the cellular mechanism for any changes in MEMRI after systemic Mn^2+^ administration. However, tracing studies that use Mn^2+^ to measure changes in axonal transport have been more straightforward. MEMRI has been used to assess pharmacological treatments in animal models of neurodegeneration and can serve as a screening tool to identify potential therapeutics. Development of MRI agents that can be used at safe doses or use of PET manganese radiotracers might enable the translation of these exciting results to humans.

## Author Contributions

GS wrote the manuscript. AK proposed the topic and edited the manuscript.

## Conflict of Interest Statement

The authors declare that the research was conducted in the absence of any commercial or financial relationships that could be construed as a potential conflict of interest.
